# Interplay between the genetics of personality traits, severe psychiatric disorders and COVID-19 host genetics in the susceptibility to SARS-CoV-2 infection

**DOI:** 10.1192/bjo.2021.1030

**Published:** 2021-10-07

**Authors:** Urs Heilbronner, Fabian Streit, Thomas Vogl, Fanny Senner, Sabrina K. Schaupp, Daniela Reich-Erkelenz, Sergi Papiol, Mojtaba Oraki Kohshour, Farahnaz Klöhn-Saghatolislam, Janos L. Kalman, Maria Heilbronner, Katrin Gade, Ashley L. Comes, Monika Budde, Till F. M. Andlauer, Heike Anderson-Schmidt, Kristina Adorjan, Til Stürmer, Adrian Loerbroks, Manfred Amelang, Eric Poisel, Jerome Foo, Stefanie Heilmann-Heimbach, Andreas J. Forstner, Franziska Degenhardt, Jörg Zimmermann, Jens Wiltfang, Martin von Hagen, Carsten Spitzer, Max Schmauss, Eva Reininghaus, Jens Reimer, Carsten Konrad, Georg Juckel, Fabian U. Lang, Markus Jäger, Christian Figge, Andreas J. Fallgatter, Detlef E. Dietrich, Udo Dannlowski, Bernhardt T. Baune, Volker Arolt, Ion-George Anghelescu, Markus M. Nöthen, Stephanie H. Witt, Ole A. Andreassen, Chi-Hua Chen, Peter Falkai, Marcella Rietschel, Thomas G. Schulze, Eva C. Schulte

**Affiliations:** Institute of Psychiatric Phenomics and Genomics (IPPG), University Hospital, University of Munich, Munich, Germany; Department of Genetic Epidemiology in Psychiatry, Central Institute of Mental Health, Medical Faculty Mannheim, University of Heidelberg, Germany; Institute of Psychiatric Phenomics and Genomics (IPPG), University Hospital, University of Munich, Munich, Germany; Institute of Psychiatric Phenomics and Genomics (IPPG), University Hospital, University of Munich, Munich, Germany; and Department of Psychiatry and Psychotherapy, University Hospital, University of Munich, Munich, Germany; Institute of Psychiatric Phenomics and Genomics (IPPG), University Hospital, University of Munich, Munich, Germany; Institute of Psychiatric Phenomics and Genomics (IPPG), University Hospital, University of Munich, Munich, Germany; and Department of Psychiatry and Psychotherapy, University Hospital, University of Munich, Munich, Germany; Institute of Psychiatric Phenomics and Genomics (IPPG), University Hospital, University of Munich, Munich, Germany; and Department of Immunology, School of Medicine, Ahvaz Jundishapur University of Medical Sciences, Iran; Institute of Psychiatric Phenomics and Genomics (IPPG), University Hospital, University of Munich, Munich, Germany; and Department of Psychiatry and Psychotherapy, University Hospital, University of Munich, Munich, Germany; Institute of Psychiatric Phenomics and Genomics (IPPG), University Hospital, University of Munich, Munich, Germany; Department of Psychiatry and Psychotherapy, University Medical Center Göttingen, Germany; Institute of Psychiatric Phenomics and Genomics (IPPG), University Hospital, University of Munich, Munich, Germany; Department of Neurology, Klinikum rechts der Isar, School of Medicine, Technical University of Munich, Germany; Department of Psychiatry and Psychotherapy, University Medical Center Göttingen, Germany; Institute of Psychiatric Phenomics and Genomics (IPPG), University Hospital, University of Munich, Munich, Germany; and Department of Psychiatry and Psychotherapy, University Hospital, University of Munich, Munich, Germany; Department of Epidemiology, Gillings School of Global Public Health, University of North Carolina at Chapel Hill, USA; Institute of Occupational, Social, and Environmental Medicine, Centre for Health and Society, Faculty of Medicine, University of Düsseldorf, Germany; Department of Psychology, University of Heidelberg, Germany; Department of Genetic Epidemiology in Psychiatry, Central Institute of Mental Health, Medical Faculty Mannheim, University of Heidelberg, Germany; Institute of Human Genetics, University of Bonn School of Medicine & University Hospital Bonn, Germany; Institute of Human Genetics, University of Bonn School of Medicine & University Hospital Bonn, Germany; and Institute of Neuroscience and Medicine (INM-1), Research Center Jülich, Germany; Institute of Human Genetics, University of Bonn School of Medicine & University Hospital Bonn, Germany; and Department of Child and Adolescent Psychiatry, Psychosomatics and Psychotherapy, University Hospital Essen, University of Duisburg-Essen, Germany; Psychiatrieverbund Oldenburger Land gGmbH, Karl-Jaspers-Klinik, Germany; Department of Psychiatry and Psychotherapy, University Medical Center Göttingen, Germany; Clinic for Psychiatry and Psychotherapy, Clinical Center Werra-Meißner, Germany; Department of Psychosomatic Medicine and Psychotherapy, University Medical Center Rostock, Germany; Department of Psychiatry, Psychotherapy and Psychosomatics, Augsburg University, Medical Faculty, Germany; Department of Psychiatry and Psychotherapeutic Medicine, Research Unit for Bipolar Affective Disorder, Medical University of Graz, Austria; Department of Psychiatry and Psychotherapy, University Medical Center Hamburg-Eppendorf, Germany; Department of Psychiatry and Psychotherapy, Agaplesion Diakonieklinikum, Germany; Department of Psychiatry, Ruhr University Bochum, LWL University Hospital, Germany; Department of Psychiatry II, Ulm University, Bezirkskrankenhaus Günzburg, Germany; Karl-Jaspers Clinic, European Medical School Oldenburg-Groningen, Germany; Department of Psychiatry and Psychotherapy, Tübingen Center for Mental Health, University Tübingen, Germany; AMEOS Clinical Center Hildesheim, Germany; and Center for Systems Neuroscience Hannover, Germany; Institute for Translational Psychiatry, University of Münster, Germany; Department of Psychiatry, University of Münster, Germany; Department of Psychiatry, Melbourne Medical School, The University of Melbourne, Australia; and The Florey Institute of Neuroscience and Mental Health, The University of Melbourne, Australia; Institute for Translational Psychiatry, University of Münster, Germany; Department of Psychiatry and Psychotherapy, Mental Health Institute Berlin, Germany; Institute of Human Genetics, University of Bonn School of Medicine & University Hospital Bonn, Germany; Department of Genetic Epidemiology in Psychiatry, Central Institute of Mental Health, Medical Faculty Mannheim, University of Heidelberg, Germany; NORMENT Centre and KG Jebsen Centre for Neurodevelopmental disorders, Institute of Clinical Medicine, University of Oslo, Norway; and Division of Mental Health and Addiction, Oslo University Hospital, Norway; Department of Radiology, University of California, USA; Department of Psychiatry and Psychotherapy, University Hospital, University of Munich, Munich, Germany; Department of Genetic Epidemiology in Psychiatry, Central Institute of Mental Health, Medical Faculty Mannheim, University of Heidelberg, Germany; Institute of Psychiatric Phenomics and Genomics (IPPG), University Hospital, University of Munich, Munich, Germany; Department of Psychiatry and Psychotherapy, University Medical Center Göttingen, Germany; Department of Psychiatry and Behavioral Sciences, Johns Hopkins University, USA; and Department of Genetic Epidemiology in Psychiatry, Central Institute of Mental Health, Medical Faculty Mannheim, University of Heidelberg, Germany; Institute of Psychiatric Phenomics and Genomics (IPPG), University Hospital, University of Munich, Munich, Germany; Department of Psychiatry and Psychotherapy, University Hospital, University of Munich, Munich, Germany; and Institute of Virology, Technical University Munich/Helmholtz Zentrum München, Germany

**Keywords:** COVID-19, extraversion, severe mental disorders, personality traits, genetics

## Abstract

**Background:**

The severe acute respiratory syndrome coronavirus 2 (SARS-CoV-2) pandemic, with its impact on our way of life, is affecting our experiences and mental health. Notably, individuals with mental disorders have been reported to have a higher risk of contracting SARS-CoV-2. Personality traits could represent an important determinant of preventative health behaviour and, therefore, the risk of contracting the virus.

**Aims:**

We examined overlapping genetic underpinnings between major psychiatric disorders, personality traits and susceptibility to SARS-CoV-2 infection.

**Method:**

Linkage disequilibrium score regression was used to explore the genetic correlations of coronavirus disease 2019 (COVID-19) susceptibility with psychiatric disorders and personality traits based on data from the largest available respective genome-wide association studies (GWAS). In two cohorts (the PsyCourse (*n* = 1346) and the HeiDE (*n* = 3266) study), polygenic risk scores were used to analyse if a genetic association between, psychiatric disorders, personality traits and COVID-19 susceptibility exists in individual-level data.

**Results:**

We observed no significant genetic correlations of COVID-19 susceptibility with psychiatric disorders. For personality traits, there was a significant genetic correlation for COVID-19 susceptibility with extraversion (*P* = 1.47 × 10^−5^; genetic correlation 0.284). Yet, this was not reflected in individual-level data from the PsyCourse and HeiDE studies.

**Conclusions:**

We identified no significant correlation between genetic risk factors for severe psychiatric disorders and genetic risk for COVID-19 susceptibility. Among the personality traits, extraversion showed evidence for a positive genetic association with COVID-19 susceptibility, in one but not in another setting. Overall, these findings highlight a complex contribution of genetic and non-genetic components in the interaction between COVID-19 susceptibility and personality traits or mental disorders.

## Background

The global spread of severe acute respiratory syndrome coronavirus 2 (SARS-CoV-2) has revealed differences in susceptibility to and severity of SARS-CoV-2 infection at both the individual and the community level. Studies from different regions of the world suggest a rise in the incidence of psychiatric disorders because of the threat of the virus and the socioeconomic repercussions of preventative measures that have been implemented.^1–3^ Interestingly, a recent study observed that a psychiatric diagnosis prior to SARS-CoV-2 infection was significantly associated with a higher risk of coronavirus disease 2019 (COVID-19) diagnosis;^4^ this risk was independent of known physical risk factors and living conditions.

Personality traits (i.e. relative stable patterns of feelings, thoughts and behaviour) might influence disease risk by mediating health-related behaviours such as the adherence to health regulations and recommendations (for example social distancing or mask wearing). In line with this, studies support an inverse relationship between extroversion and likelihood of engaging in social distancing behaviour at the beginning of the pandemic.^5,6^

The genetic underpinnings of psychiatric traits are known to not only show a large overlap among each other^7^ but also with other diseases such as metabolic disorders.^8,9^ An increased overall load of infections in individuals with psychiatric disorders has also been reported and may, in part, be because of shared genetic liability,^10^ although only few large-scale studies have tried to answer this question to date. In addition to many other factors ranging from gender to pre-existing medical conditions and socioeconomic factors,^11,12^ both common and rare genetic variants have been identified that may predispose individuals to an infection with SARS-CoV-2 or a severe course of COVID-19.^13–15^

A recent GWAS by the COVID-19 Host Genetics Initiative,^16^ identified 13 loci of genome-wide significance for susceptibility to COVID-19, comparing participants with a self- or physician-reported COVID-19 diagnosis with the general population. Four of these loci seem to be specific to COVID-19 susceptibility rather than disease severity. The identified loci include variants in genes implicated in the innate immune response to viruses but also genomic loci harbouring many genes of yet-undetermined function in the context of COVID-19.

## Aims

In light of these findings, we asked whether genetic underpinnings are shared between COVID-19 susceptibility, major psychiatric disorders and personality traits. We approached this question using both results from the largest GWAS in the respective fields and individual-level data from two observational studies of psychiatric disorders (PsyCourse) and personality traits (PsyCourse and HeiDE).

## Method

We performed linkage disequilibrium score regression (LDSC)^17^ to calculate genetic correlations^18^ between susceptibility to COVID-19 and psychiatric disorders as well as personality traits. We used summary statistics for COVID-19 susceptibility derived from a GWAS performed by the COVID-19 Host Genetics Initiative^16^ (self- or physician-reported COVID-19 diagnosis (*n* = 87 870) versus general population (*n* = 2 210 804); analysis ‘C2’ for European ancestry without 23andMe, Inc, release 6, downloaded from https://www.covid19hg.org/results/r6/, accessed 30 July 2021).

For psychiatric disorders, summary data from the following GWAS were used: schizophrenia (SCZ; 33 640 cases; 43 456 controls),^19^ bipolar disorder (BPD; 41 917 cases; 371 549 controls),^20^ depression (as a broader phenotype closely related to major depressive disorder (MDD), 246 363 cases; 561 190 controls)^21^ and Big 5 personality traits (*n* = 70 000 to 120 000).^22^ For the details on phenotype definitions used in the GWAS, please refer to the original publications.

In a second step, individual-level data were used to calculate polygenic risk scores (PRS). In the PsyCourse study (*n* = 1786), consisting of individuals with major psychiatric disorders (652 SCZ, 567 BPD, 101 MDD) and controls without major psychiatric disorders (*n* = 466), recruited throughout Germany and Austria and followed longitudinally,^23^ we assessed whether PRS for susceptibility to COVID-19 were associated with case status or with extraversion scores. PRS were calculated using the PRS-CS method,^24^ excluding the human leukocyte antigen (HLA) region on chromosome 6.

All genotyped participants of the PsyCourse study with a diagnosis from the psychotic-to-affective spectrum as well as controls (*n* = 1346, age mean  47.75, s.d. = 13.81, 47.39% female) or for whom an extraversion score was available (*n* = 1190) were included in the analysis. ‘Case’ status was defined as having a lifetime diagnosis of a severe psychiatric disorder from the spectrum of psychotic and affective disorders defined in the DSM-IV^25^ and as determined by a trained rater administering the relevant section of the SCID-I^26^ interview.

**Fig. 1:**
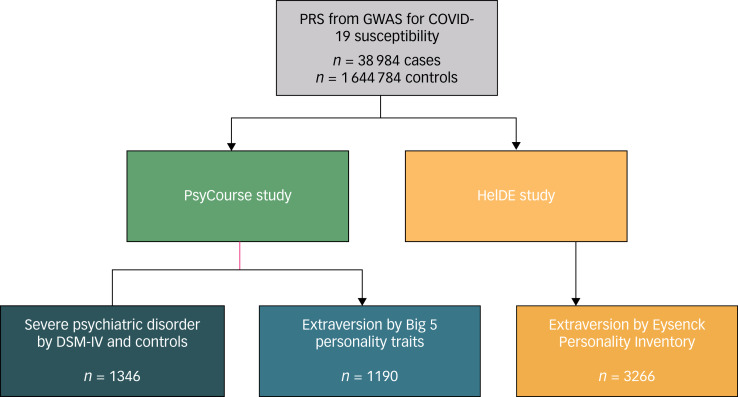
Study design for polygenic risk scores (PRS) analyses. Summary statistics from the largest genome-wide association study (GWAS) on susceptibility to coronavirus disease 2010 (COVID-19) to date performed by the COVID-19 Host Genetics Initiative were used to generate PRS. These were then tested for association with case–control status for severe psychiatric disorders such as schizophrenia, bipolar disorder or major depressive disorder (PsyCourse study only) as well as measures of extraversion (PsyCourse and HeiDE studies).

The extraversion score (range: 1 to 5, mean: 3.09) was derived from a 10-item questionnaire assessing the Big 5 personality traits^27^ (Fig. 1). DNA samples of PsyCourse participants were genotyped on the Illumina Infinium PsychArray, and imputed using the 1000 Genomes project data-set as reference panel (for details, see^23^).

In the HeiDE study (for example^28^), we assessed whether extraversion scores (see below) were associated with PRS for COVID-19 susceptibility (generated using PRS-CS; *n* = 3266, age mean 52.78, s.d. = 7.06, 52.38% female). Briefly, HeiDE (‘Heidelberger Langzeitstudie zu Risikofaktoren und Diagnose chronischer Erkrankungen’) is a population-based study carried out in the German city of Heidelberg and surrounding area with an initial aim of characterising associations of personality and somatic disease. Data analysed in this study were collected during the baseline assessment (personality traits; 1992 to 1994) and the first follow-up (DNA for genotyping; on average 8.5 years after baseline). Extraversion was measured using the Eysenck Personality Inventory^29^, from which we analysed the sum of two items closely matching the items of the Big 5 personality questionnaire used in the PsyCourse study (range: 0 to 2, mean: 1.48). DNA samples of HeiDE participants were genotyped using the Illumina Infinium PsychArray and the Infinium OmniExpress Exome Array. The combined HeiDE data-sets were imputed using the 1000 Genomes phase 3 reference panel (for details, see^30^). Also see the figure for an overview of the study design.

PRS scoring and association testing using linear or ordinal regressions were implemented in PLINK (version 1.9) and R (version 4.0.3). In both studies, we regressed the respective phenotype onto age, age^2^, gender and the first eight ancestry multidimensional scaling components (backward stepwise regression). The residuals of the final model were then regressed onto the PRS (PsyCourse), or the final model was compared with a model additionally containing the PRS (HeiDE). As far as we know, there is no overlap between individuals from the PsyCourse and HeiDE studies and the GWAS, whose summary statistics were used for the LDSC above.

The authors assert that all procedures contributing to this work comply with the ethical standards of the relevant national and institutional committees on human experimentation and with the Helsinki Declaration of 1975, as revised in 2008. All procedures involving human participants were approved by the Institutional Review Board at the University of Munich,^13–17^ the Institutional Review Board at the Medical Faculty of the University of Heidelberg (026/2001), or the local review boards of the primary studies that the utilised summary statistics were taken from.^16,19–22,31^ Written informed consent was obtained from all study participants.

## Results

No genetic correlation was found between COVID-19 susceptibility and MDD, BPD, or SCZ risk (Table 1).

**Table 1:** Results from linkage disequilibrium score regression between coronavirus disease 2019 susceptibility and severe psychiatric disorders

Trait	Major depressive disorder^21^	Bipolar disorder^20^	Schizophrenia^19^
Genetic correlation	0.072	0.004	−0.037
Standard error	0.044	0.045	0.044
*P*	0.100	0.930	0.398

When analysing the genetic correlation between personality traits^22^ and COVID-19 susceptibility, a significant positive correlation (*P* = 1.47 × 10^−5^; genetic correlation 0.284) was identified for the personality trait of extraversion. No statistically significant correlation was present with any other Big-5 personality trait (Table 2).

**Table 2:** Results from linkage disequilibrium score regression between coronavirus disease 2019 (COVID-19) susceptibility and Big 5 personality traits^a^

Trait	Agreeableness	Conscientiousness	Extraversion	Neuroticism	Openness
Genetic correlation	0.177	0.056	0.284	−0.105	−0.095
Standard error	0.093	0.079	0.066	0.071	0.067
*P*	0.057	0.478	1.47 × 10^−5^	0.142	0.153

a. The *P* for genetic correlation between extraversion and COVID-19 susceptibility was statistically significant.

To corroborate these findings with a second, independent line of evidence using individual-level data from two independent cohorts, we turned to an assessment of PRS. In the PsyCourse study, PRS for COVID-19 susceptibility were not significantly associated with psychiatric case status when compared with controls (*P* = 0.474, beta = −1.132). Further, in the PsyCourse study, no significant association between COVID-19 susceptibility PRS and extraversion as measured by the 10-item questionnaire assessing the Big-5 personality traits (*P* = 0.210, beta = 2.369) was found.

To validate the finding for extraversion in another study setting and to mitigate any potential influence of an interaction between psychiatric disorders and personality traits in the context of COVID-19 susceptibility possibly present in the PsyCourse study, we recapitulated the extraversion analysis in the larger HeiDE study, which was specifically designed to evaluate the interaction between personality traits and somatic disorders. Here, however, we also did not detect a significant association for PRS for COVID-19 susceptibility and extraversion (model comparison *P* = 0.758, AIC 5149.51 (model with PRS) and AIC 5147.60 (model without PRS)).

## Discussion

### Main findings

It is likely that many interdependencies exist between COVID-19 susceptibility and major psychiatric disorders or personality traits. Among these, we shed light on a potential role for shared common genetic risk factors. For major psychiatric disorders, we did not identify a significant genetic overlap that can be ascribed to common genetic variation both when assessing summary statistics of large GWAS by LDSC and when looking at PRS in individual-level data, in line with emerging data in the field.^16,32,33^

With regard to personality traits, the picture is more heterogeneous with a significant signal for a positive genetic correlation between extraversion and COVID-19 susceptibility by LDSC, which needs to be explored further once larger data-sets become available. However, it has to be assumed that the genetic make-up is only one contributor in a very complex network of factors connecting extraversion to COVID-19 susceptibility.

### Interpretation of our findings and comparison with findings from other studies

The positive correlation identified between COVID-19 susceptibility and extraversion highlighted by the LDSC approach appears to be in line with the literature. Numerous studies performed both before and during the SARS-CoV-2 pandemic have demonstrated the effect of personality determinants on health behaviour and outcomes (such as ^5,34,35^). For example, it was shown that narcissistic tendencies coincide with decreased perceived susceptibility to infection with SARS-CoV-2^36^ whereas, at least for neuroticism, no genetic overlap was found.^32^ Intuitively, less extroverted individuals may find social distancing during the pandemic easier than extroverted individuals and may, therefore, be more compliant with social distancing rules and at an overall decreased risk of COVID-19.^5,6^

There is even evidence of a bidirectionality of this phenomenon – the general risk for infectious diseases in a given region may, in part, influence personality traits at population level such that lower mean levels of extraversion are reported in regions with higher prevalence of infectious diseases.^37^ One possible reason for this could be that in regions where ever-present infectious diseases present a comparatively large threat to health and well-being, less extraversion is present at population level either because people have adapted their behaviour or because of potential selective pressure. Yet, it is likely that many interdependencies exist between COVID-19 susceptibility and personality traits or major psychiatric disorders and we investigated only shared common genetic risk factors.

### Limitations

Although all included GWAS are the currently largest in the respective fields, sample sizes may still not be large enough to confidently detect genetic correlations in settings with many natural confounders such as levels of exposure to the virus or socioeconomic differences, to name only a few. Also, different instruments were used to evaluate personality traits in PsyCourse and HeiDE and the study populations (individuals with severe psychiatric disorders and controls versus the general population) were different, possibly contributing to the observed heterogeneity.

Although LDSC represents a powerful tool to assess genetic correlations, other methods to quantify polygenic overlap irrespective of genetic correlations also exist (for example ^38^) and could be used to explore potential shared genetic underpinnings in even greater depth but are beyond the scope of this study. An additional limitation lies in the fact that no direct risk assessment was possible for the individuals with individual-level data on major psychiatric disorders and personality traits since no COVID-19 phenotypes were available. Finally, we are unable to fully exclude sample overlap especially for the controls used in the included GWAS. However, LDSC results should be robust to this overlap.^18^

### Implications

Hypothetically, it is possible that – for example – only a small subset of common genetic risk factors in a given pathway relevant to major psychiatric disorders or personality traits is associated with COVID-19 susceptibility. Although we cannot fully exclude all such effects, our data suggest that non-genetic factors play important roles in the interplay between personality traits and COVID-19.

A direct genetic overlap is unlikely to contribute to the increased, but yet-unexplained COVID-19 risk seen in individuals with a psychiatric diagnosis prior to SARS-CoV-2 infection^4^ but a shared genetic risk could still be mediated by intermediate phenotypes such as, for example, lower socioeconomic status or educational attainment in those with severe psychotic disorders. As a consequence, an even greater focus should be placed on psychosocial interventions, ensuring the best treatment for individuals with severe psychiatric disorders as well as targeted measures of prevention and psychoeducation for individuals with personality determinants that place them at an increased pandemic-related risk for health and well-being.

## Data Availability

The data that support the findings in this study are available from the corresponding author, E.C.S., upon reasonable request. The relevant summary statistics from the GWAS used in the analyses are available from the authors of the primary studies.^16,19–22,31^ Interested researchers can also apply for the used as well as additional data for the PsyCourse study via http://www.psycourse.de/openscience-en.html.
