# Interplay between the Genetics of Personality Traits, severe Psychiatric Disorders, and COVID-19 Host Genetics in the Susceptibility to SARS-CoV-2 Infection - ADDENDUM

**DOI:** 10.1192/bjo.2021.1037

**Published:** 2021-11-18

**Authors:** Urs Heilbronner, Fabian Streit, Thomas Vogl, Fanny Senner, Sabrina K. Schaupp, Daniela Reich-Erkelenz, Sergi Papiol, Mojtaba Oraki Kohshour, Farahnaz Klöhn-Saghatolislam, Janos L. Kalman, Maria Heilbronner, Katrin Gade, Ashley L. Comes, Monika Budde, Till F. M. Andlauer, Heike Anderson-Schmidt, Kristina Adorjan, Til Stürmer, Adrian Loerbroks, Manfred Amelang, Eric Poisel, Jerome Foo, Stefanie Heilmann-Heimbach, Andreas J. Forstner, Franziska Degenhardt, Jörg Zimmermann, Jens Wiltfang, Martin von Hagen, Carsten Spitzer, Max Schmauss, Eva Reininghaus, Jens Reimer, Carsten Konrad, Georg Juckel, Fabian U. Lang, Markus Jäger, Christian Figge, Andreas J. Fallgatter, Detlef E. Dietrich, Udo Dannlowski, Bernhardt T. Baune, Volker Arolt, Ion-George Anghelescu, Markus M. Nöthen, Stephanie H. Witt, Ole A. Andreassen, Chi-Hua Chen, Peter Falkai, Marcella Rietschel, Thomas G. Schulze, Eva C. Schulte

An affiliation was missed for Thomas G. Schulze. The missed affiliation was for the Department of Psychiatry and Behavioral Sciences, SUNY Upstate Medical University, Syracuse, NY, USA. The Publisher apologises for this omission.
